# MRI of Paratubal Borderline Serous Tumor: Teaching point: Micropapillary projections in a thin walled cystic mass located along a salpinx are suggestive of a paratubal borderline serous tumor

**DOI:** 10.5334/jbsr.1618

**Published:** 2019-08-13

**Authors:** Eveline Claus, Koen Van de Vijver, Pieter De Visschere

**Affiliations:** 1 UZ Gent, BE

**Keywords:** paratubal serous borderline tumor

## Case report

**Figure 1:**
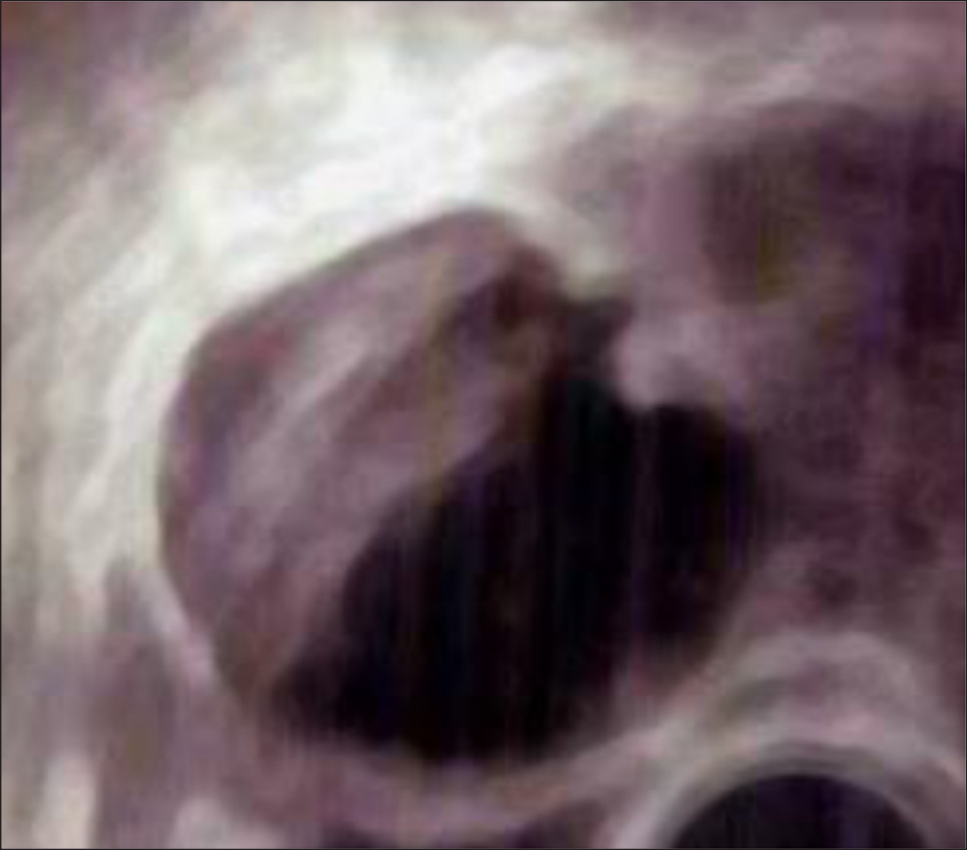
Ultrasound.

**Figure 2:**
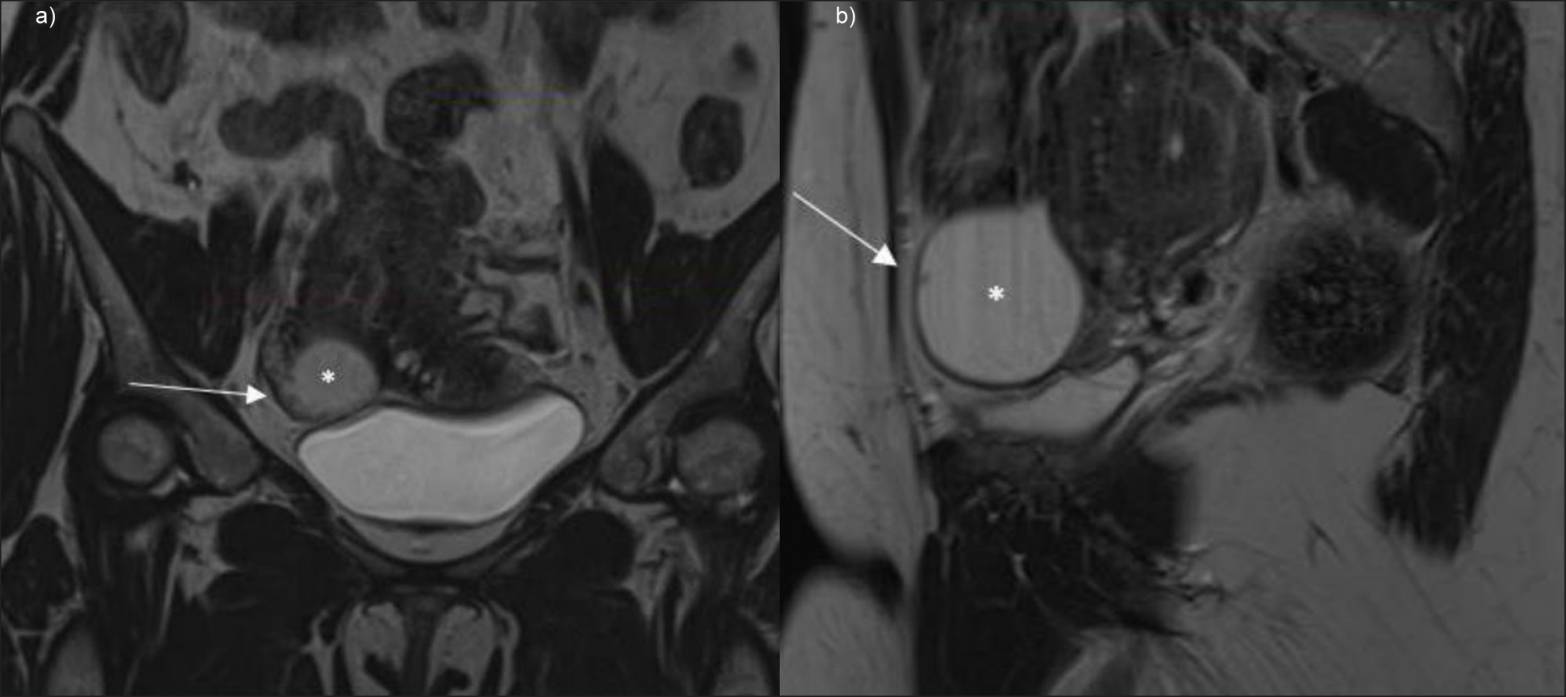
T2-weighted MR (**a**, coronal, left panel; **b**, parasagittal, right panel).

**Figure 3:**
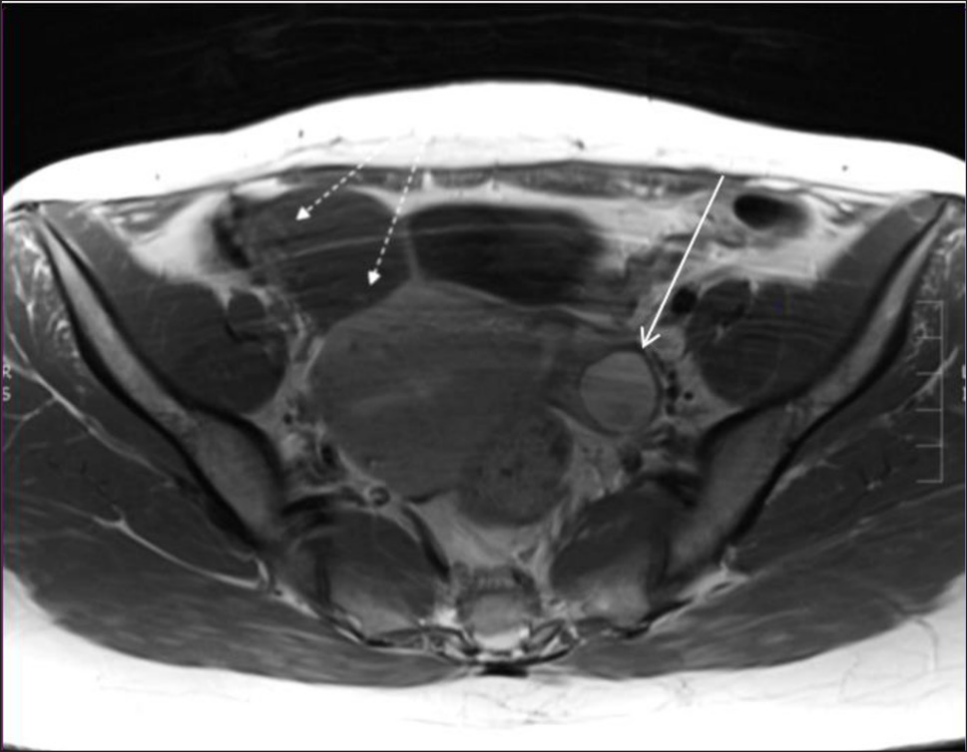
Axial contrast-enhanced T1-weighted MR.

A 40-year-old woman was admitted to the hospital because she complained of lower abdominal pain. An ultrasound performed by the gynecologist (Figure 1) showed an adnexal thin walled cyst of 4 cm diameter in the right fossa with multiple tiny papillary excrescences on the internal wall. CA-125 was slightly elevated (65 kU/L). On magnetic resonance (MR), the cyst showed homogenous high signal intensity on T2-weighted imaging (white star on Figure 2) and the numerous very small excrescences in the wall (arrow on Figure 2) were confirmed. It showed homogenous low signal intensity on T1-weighted imaging and numerous very small (millimetric) spots of contrast enhancement in the wall (white dashed arrow on Figure 3). The cyst appeared to originate from the right salpinx, at a distance from the right ovary. An incidental hemorrhagic cyst was seen in the left ovary (thick arrow on Figure 3).

The cyst was surgically resected, and histopathology revealed a paratubal borderline serous tumor (atypical proliferative serous tumor, APST) originating from the vesicular appendage of the right epoöphoron.

## Comment

Tubal and paratubal serous borderline tumors (SBT) are of Müllerian origin. Though they are not limited to a specific part of the fallopian tube, ovarian SBT are common, whereas their fallopian tubal and paratubal counterparts are rare. To our knowledge, only two cases of paratubal SBT have been described in the literature. We add a third case and describe its appearances on MR for the first time. These characteristics are similar to that of their ovarian counterparts. In our case, some tiny enhancing micropapillary protrusions could be distinguished. This is compatible with the peroperative finding of papillary excrescences in the wall of the cyst, as described by Seamon et al. [1]. Histopathology shows a cyst with irregular papillae in a hierarchical branching pattern, lined by a pseudostratified single layer of columnar epithelium. SBT are distinguished from benign tumors by epithelial budding, increased mitotic activity, and mild nuclear atypia. Thus, picking these excrescences on imaging seems quite specific, but further imaging and reporting of these rare tumors is necessary. No further distinctive traits on imaging are mentioned, and in all previous cases that were diagnosed on histopathology.

Microscopically, all Müllerian SBT are identical [1], although paratubal SBT are sometimes regarded as peritoneal SBT by pathologists. Due to the rarity of these tumors, little is known about the clinical behavior and the optimal treatment of these tumors. Surgical resection of the cyst is usually performed, with or without partial salpingectomy. Fertility-sparing surgery may be considered in patients who wish to preserve childbearing potential. Salpingectomy and comprehensive staging should be performed immediately if a frozen section shows a borderline tumor, to avoid a second operation if the final diagnosis turns out to be invasive carcinoma.
